# A Tensioned Human Skin Explant Model Used for Preliminary Assessment of Chemexfoliant-Stimulated Bioeffects

**DOI:** 10.1016/j.xjidi.2024.100305

**Published:** 2024-08-23

**Authors:** Michael J. Conneely, Jin Namkoong, Francis Allison, S. Kyoko Hirata Tsutsumi, Dominic Grussu, Ryan Willis, Kyle Henderson, Paul A. Campbell, Melissa Moy, Ewelina Lesniak, Joanna Wu, Robyn P. Hickerson

**Affiliations:** 1 Ten Bio, Dundee, Scotland; 2 Research and Innovation, Department of Global Personal Care and Skin Health R&D, Colgate-Palmolive, Piscataway, New Jersey, USA; 3 Biological Chemistry and Drug Discovery, School of Life Sciences, University of Dundee, Dundee, Scotland; 4 PAUL, Carnegie Physics Laboratory, University of Dundee, Dundee, Scotland; 5 Personal Care Product Development, Skin Health R&D, Colgate-Palmolive, Piscataway, New Jersey, USA

**Keywords:** Chemexfoliation, Chemical peeling, Ex vivo skin explant, Skin rejuvenation, Tension

## Abstract

A tensioned ex vivo full-thickness human skin explant platform was used to assess the bioeffects arising from application of several commercial chemexfoliation agents. Although such treatments are well-established, and improved understanding of the underlying mechanistic processes continues to emerge, research into the optimum treatments for specific skin types/conditions is still needed for enhanced efficacy while minimizing recovery time. The 3 commercial chemexfoliation agents employed all contained trichloroacetic acid at well-defined concentrations (6, 10, and 20%) and were applied to the explants’ stratum corneum. Subsequently, measurements of dermal remodeling factors (COL1A1, ELN, HAS2, HAS3, and procollagen type I) and inflammatory marker (IL-1b) were undertaken using qPCR and immunofluorescent analyses. Statistical analysis of these data facilitated the establishment of benchmarking biological responses to these trichloroacetic acid–containing agents against untreated controls. The performance of an innovative trichloroacetic acid–free chemexfoliation agent was then measured and, upon comparison with the previous benchmarking data, indicated that dermal remodeling factors could be upregulated in fashion comparable with that of the trichloroacetic acid–containing agents but with significant suppression of inflammatory response. Our measurements thus underscore the promise of the tensioned explant over prolonged study periods and also that potentially valuable insights to guide preclinical strategies may be forthcoming from the protocol developed.

## Introduction

Methods to maintain or enhance the appearance of skin have been known since ancient times (Bryan and Smith, 1930). Of the methodologies in use presently (Weissler et al, 2017), chemexfoliation (chemical peeling) has emerged as a popular mainstream approach owing to the minimally invasive nature of the procedure, the relatively low costs involved, and the wide variety of skin conditions that can be successfully treated through the approach (Conforti et al, 2020; Weissler et al, 2017). As such, the breadth of esthetic and, indeed, therapeutic applications that involve chemexfoliation have led to a significant and expanding global market value for that particular treatment, with estimated revenues exceeding $2 billion (Statista, 2024).

In essence, chemical peeling involves the topical application of a chemexfoliation agent, the identity and concentration of which must be tailored to the distinct skin type, cosmetic need, or clinical objective. Chemical peels that penetrate only into the epidermis (defined as a superficial peel) result in reduction/removal of the stratum corneum and lead to regeneration and thickening of the epidermis. Of the common chemexfoliants that readily achieve superficial peels, several are pertinent to this study. For example, lactic acid is typical of the water soluble alpha hydroxy acids and is commonly used to treat conditions such as actinic keratoses, solar lentigines, mild acne scars, and certain pigmentary disorders. The mechanism of action principally involves corneocyte desquamation (Fartasch et al, 1997; Rakic et al, 2000). Similar presentations may be alternatively treated using the oil-soluble beta hydroxy acids, of which salicylic acid is a prevalent example. In this case, exfoliation of predominantly oily skin is achieved by the initial agent penetration into the sebum-rich pores (O’Connor et al, 2018). Use of more aggressive chemexfoliants facilitates deeper penetration through the epidermis and into the papillary dermis (creating a medium-depth peel), thus promoting a more pronounced response, proceeding into the reticular dermis, and stimulating collagen regeneration resulting in a rejuvenated skin appearance. Stronger chemexfoliation agents have the potential to penetrate yet deeper into subdermal structure and thus may represent a more invasive procedure unless distinct controls are exercised. The majority of chemexfoliation procedures are thus conducted by qualified professionals, with distinct procedural strategies chosen judiciously on the basis of the assessment of an individual’s skin type and the clinical or esthetic demand. Of the multitude of commercial chemexfoliation agents at the practitioners’ disposal (Conforti et al, 2020) for achieving medium-to-deep peels, those containing several commonly utilized chemical active ingredients dominate both markets and clinical choice owing to their outward efficacies and the fact that an understanding of their clinical response has been well-established. One such agent is trichloroacetic acid (TCA) (Nguyen and Rooney, 2000), also known as trichlorinated carbonic acid, commercial incarnations of which are routinely prescribed for various indications, including melasma, photoaging and the associated presentations, pigmentary disorders, seborrheic keratosis, and acne scarring. The action of TCA-based compounds was first described by Roberts (1926), and the active ingredient has retained its popularity because TCA is easily diluted to prescribed concentrations (Lee et al, 2019; O’Connor et al, 2018) that permit correct targeting at the required depth of penetration, often through a multilayered reapplications onto the skin to achieve the desired clinical outcome in the most controllable fashion.

Notably, however, use of TCA-based products can be associated with a heightened sensitivity during application as well as possible frosting if used at higher concentrations. Owing to the rapid cauterant mechanism of TCA, it cannot be readily neutralized, and therefore once applied to the skin, it continues to penetrate until its activity has been exhausted in the coagulation of protein (Rubin, 2018), which, dependent on individual donor response, can result in severe redness, stinging sensation, irritation, peeling, and prolonged recovery periods. Empirically, the outward expectation is that higher TCA concentrations lead to deeper peel penetrations. Clearly, alternative chemexfoliation agents would be advantageous if the benefits delivered were at least comparable with TCA-based peels but with reduced detrimental side effects arising. Assessing the performance of potential new agents in a preclinical context represents a secondary aim of this study. However, achieving that objective requires first that the bioeffects arising from application of traditional commercial agents be initially benchmarked to facilitate direct comparisons.

It is notable that in pursuit of this latter objective, recent directives (such as European Union, 2010) seeking to replace, reduce, and refine the use of animals used for scientific purposes have constrained skin-related product development, especially where true clinical relevance is demanded. At a minimum, a specific requirement for a useful alternative to animal models must be that they retain viability from the initial inflammation reaction (2–4 days after treatment), through to the dermal remodeling stage (approximately 2 weeks). Sophisticated in vitro cultured human skin equivalent models offer 1 promising avenue toward resolving this quandary (Meleshina et al, 2017); however, a more direct strategy involves the exploitation of ex vivo human skin platforms (explants) (Souci and Denesvr, 2021). In this study, it is realized that the outward lack of innervation, vascular perfusion, and somewhat constrained inflammatory response limit an explant’s utility as a true clinical mimic. However, it is also clear that explants, cultured under well-defined conditions, can be maintained in a viable and biologically robust state over extended periods, and their intrinsic response to stimulation and/or insult can still provide meaningful insights that serve as a useful guide for preclinical strategies. As an initial step in the latter direction, we have assessed 1 such biologically active ex vivo skin platform (TenSkin) for its suitability in conducting chemexfoliation optimization research, in preparation for downstream use in humans. The TenSkin platform holds ex vivo skin under an optimized tension condition, a factor that has been demonstrated to both enhance longevity under culture conditions beyond 14 days and replicate key aspects of normal physiological skin response, thus offering preclinical insights in a biologically robust model (Conneely et al, 2022, 2018; Szeder et al, 2022). Specifically, we exploited TenSkin to benchmark the biological response from several commercially available TCA-based chemexfoliation products (of varying TCA concentrations at 6, 10, and 20% w/w) in terms of their facility to induce dermal remodeling factors, while also quantitatively assessing any parallel inflammatory responses. This objective formed the primary goal of this study.

We extended the study to assess the performance of, to our knowledge, a previously unreported hydroxy acid–based chemexfoliant, which importantly is TCA free (and henceforth referred to as the non-TCA agent) and rather contains a combination of salicylic acid, lactic acid, and a polyhydroxy acid (gluconolactone). This non-TCA agent was designed to retain the outward esthetic and health benefits of popular present-day chemexfoliation treatments but to also mitigate against the risks and recovery time associated with such traditional chemexfoliation procedures mentioned earlier. In this study, the non-TCA formulation was designed to include a blend of traditional chemexfoliation acids with additional exfoliating botanical extracts to ensure a less aggressive peel. Moreover, by inclusion of gluconolactone, a next-generation peel ingredient within the new formulation, we wished to test the hypothesis that advantageous stimulation of remodeling factors could still be achieved, but with reduced risk of iatrogenic injury, as could be evidenced by reduced inflammatory markers. Again, qPCR was employed to measure markers of both inflammation and remodeling responses. In addition, procollagen I protein expression was evaluated using immunofluorescence staining, with the outward goal of assessing whether any signatures associated with potentially beneficial outcomes normally arising in conventional TCA-based treatments can be replicated with the non-TCA agent application, while simultaneously reducing risk factors.

## Results and Discussion

**Figure 1:**
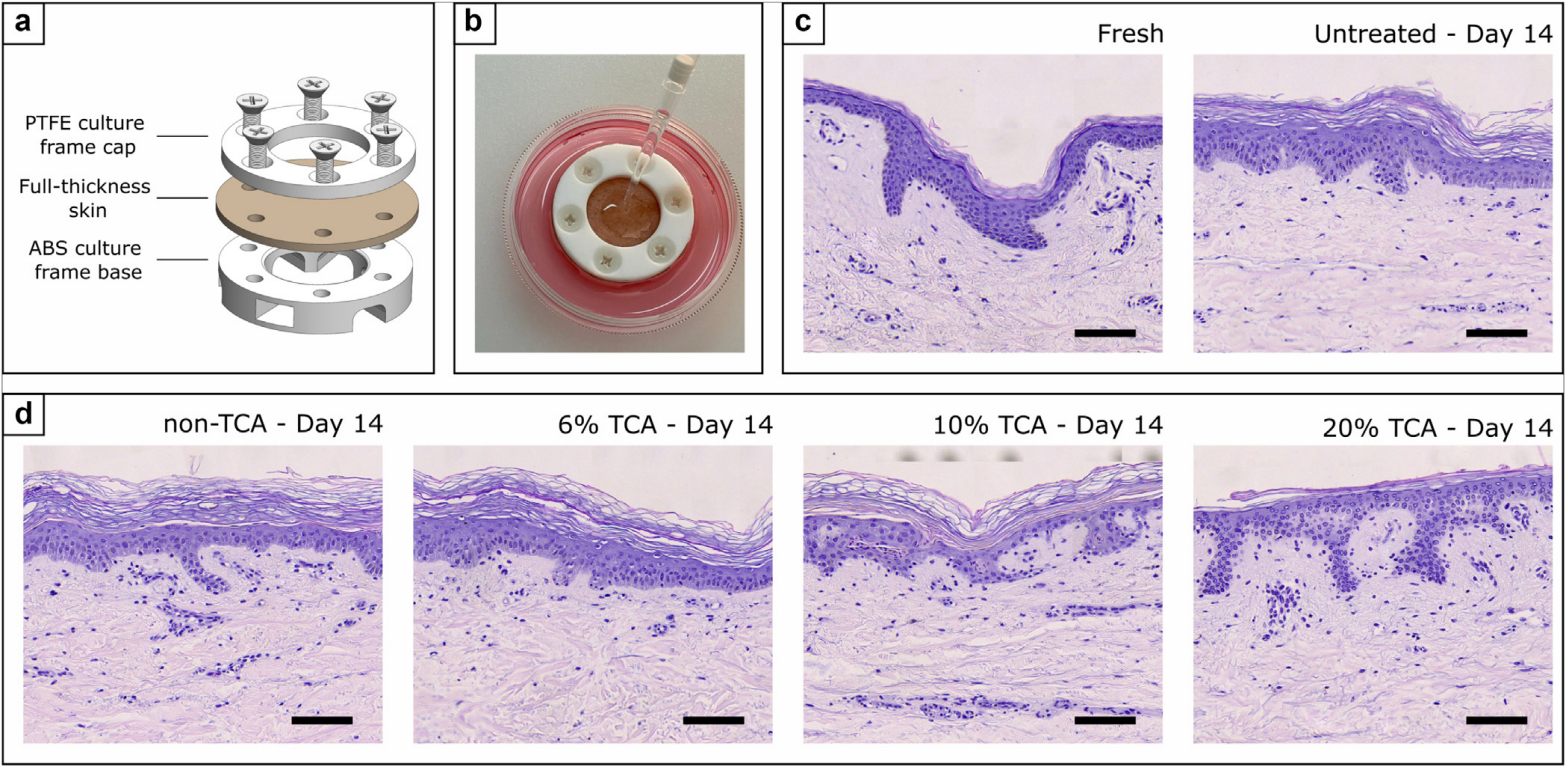
**Ex vivo platform for analysis of professional peel formulations and representative appearances before and after treatment.** (**a**) Schematic illustrating the TenSkin ex vivo platform in which full-thickness human abdominal skin is maintained at physiological tension. (**b**) Topical application of peel treatment by direct pipetting. (**c**, **d**) Representative H&E-stained sections showing integrity of tissue maintained in culture up to 14 days for each of the distinct TCA concentrations used to establish the benchmark response of TenSkin against these commercial chemexfoliation agents. Bars in **c** and **d** represent 100 μm. ABS, acrylonitrile butadiene styrene; PTFE, polytetrafluoroethylene; TCA, trichloroacetic acid; UT, untreated.

In overview, initially, the skin from 3 human donors (all of whom were White female donors and were aged 42, 43, and 59 years, respectively) was used to make multiple ex vivo human skin model platforms (TenSkin) (Figure 1a and b). The biological response of these explant models was then measured after treatment with 3 different commercially available TCA-based combination chemexfoliation agents (listed in Table 1).

**Table 1:** Combination Chemexfoliation Agent Compositions

Agent Characteristic	non-TCA Combination Peel	6% TCA Combination Peel	10% TCA Combination Peel	20% TCA Combination Peel
Main active ingredients	15% lactic acid 10% PHA (gluconolactone) 2% SA	6% TCA 12% lactic acid	10% TCA 24% lactic acid	20% TCA 10% lactic acid
pH	3.0–3.5	0.63–1.63	0.15–1.15	0.13–1.13

Abbreviations: PHA, polyhydroxy acid; SA, salicylic acid; TCA, trichloroacetic acid.

This initial evaluation exercise was conducted with the primary objective of benchmarking the explant model’s biological response to treatment using the various TCA-containing commercial agents and with assessment conducted relative to untreated controls from each individual skin donor. As such, 4 explant models developed from each donor were used in this initial benchmarking study (1 untreated control and 1 each for the 3 treatments using the 3 different TCA-containing chemexfoliation agents). After treatment, each explant then had 3 × 3 mm biopsies taken for downstream measurement of biological response using RT-qPCR. Three biopsies per explant were deemed necessary to account for natural structural inhomogeneity across the treated explants. Each of the 4 treatment regimens thus provided 9 datum points (3 biopsies from each of the 3 skin donors) for the benchmarking exercise.

**Figure 2:**
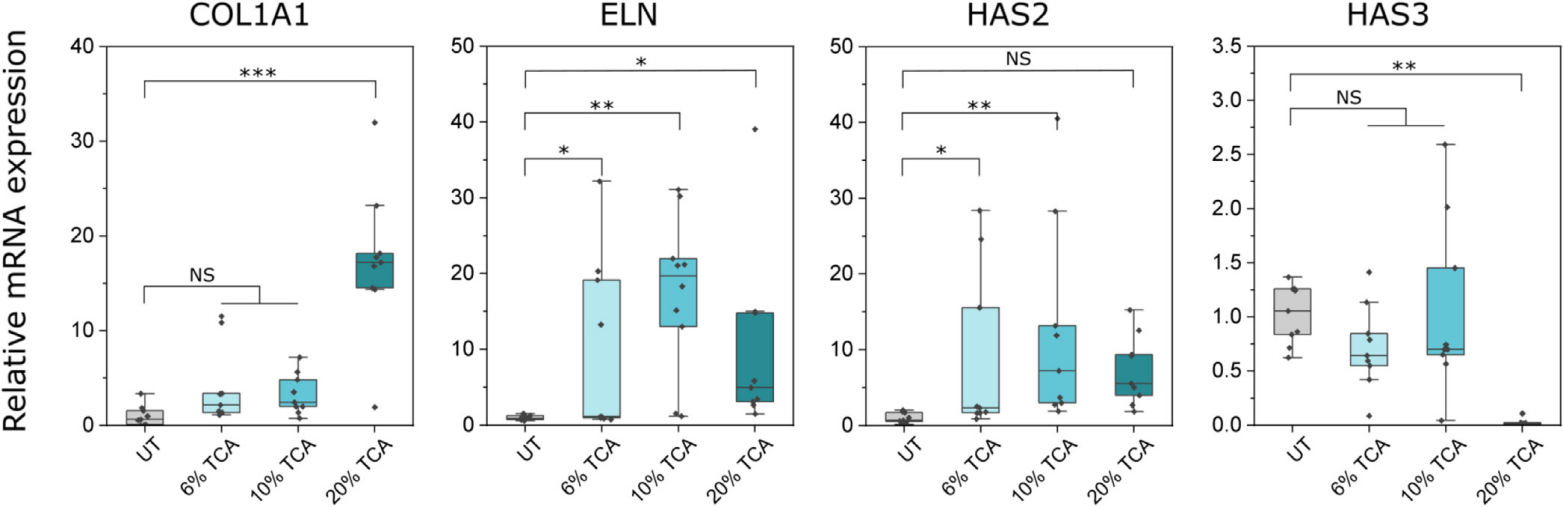
**Dermal matrix protein mRNA expression profiles in ex vivo skin obtained from RT-qPCR measurement as a function of TCA concentration levels across 3 commercial chemexfoliation agents.** Average mRNA expression from 3 donors (all White females aged 42, 43, and 59 years, respectively) is shown. Significance is displayed in the graph (∗*P* ≤ .05, ∗∗*P* ≤ .01, and ∗∗∗*P* ≤ .001 relative to the UT donors’ controls and with NS labeling indicating nonsignificant differences) as determined by a 2-way repeated measures ANOVA, and the data are presented in boxplots with overlayed dot plots from 3 biopsies from each of the 3 donors involved. The box represents the IQR, with a black line for the median value and whiskers representing values within 1.5 × IQR. IQR, interquartile range; TCA, trichloroacetic acid; UT, untreated.

These measurements are graphically summarized in Figure 2. Note that the overall scheme for the workflow is further clarified in pictorial form within the Materials and Methods section.

**Figure 3:**
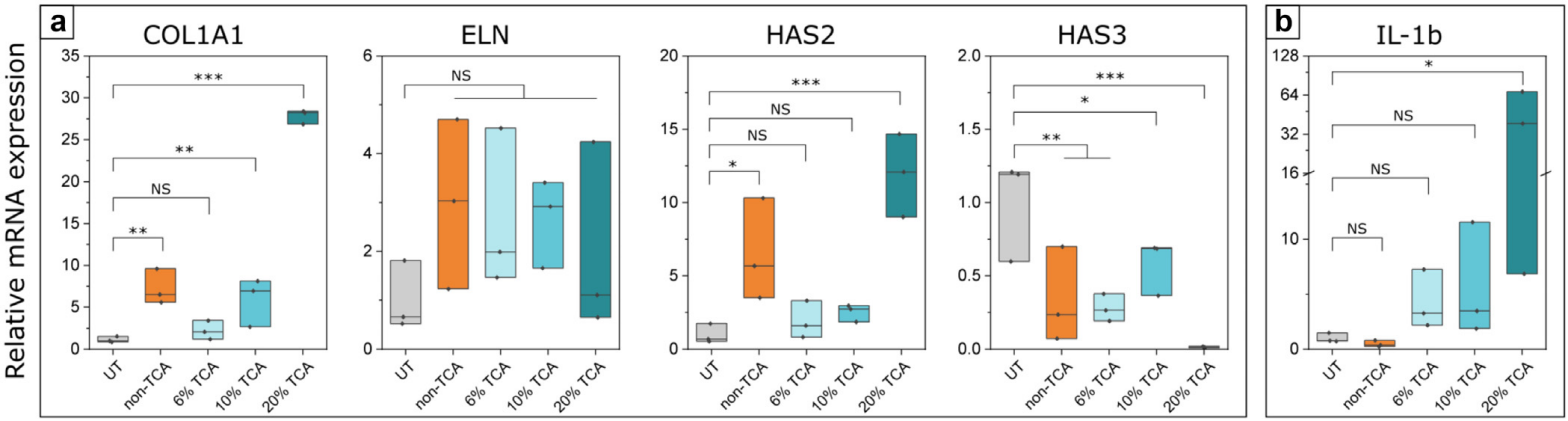
**Dermal matrix protein and inflammation mRNA expression profiles in chemexfoliant-treated ex vivo skin (White female aged 43 years) using RT-qPCR.** (**a**) Comparison of the effects of the 3 TCA-containing chemexfoliation agents (6, 10, and 20% TCA) with the non-TCA agent (composition detailed in Table 1) and the UT control 14 days after application of the treatments. (**b**) Inflammation marker IL-1b was evaluated 3 days after treatment during the peak period of the induced inflammatory cascade. Significance is displayed within each graph (∗*P* ≤ .05, ∗∗*P* ≤ .01, and ∗∗∗*P* ≤ .001 with NS labeling indicating nonsignificant differences) as determined by a 1-way repeated measures ANOVA. The data are presented as mean ± SD from 3 biopsies from each of 5 separately treated TenSkin explants, each constructed using only this particular donor’s skin. The data are presented in boxplots, with overlayed dot plots from 3 biopsies taken from the single additional donor. The box represents the IQR, with a black line for the median value. Note also that this single additional donor had not been involved in the preliminary benchmarking study presented in Figure 2. IQR, interquartile range; TCA, trichloroacetic acid; UT, untreated.

With the benchmarking exercise completed, we then proceeded to assess the performance of the additional chemexfoliation formulation (the non-TCA agent) on explants developed from a single additional skin donor. For this study, 5 explants were used from an additional (single) donor’s skin: one served as the untreated control; another was treated with the non-TCA agent; and the final 3 were treated with the TCA-containing agents again at respective concentrations of 6, 10, and 20%. Again, 3 × 3 mm biopsies were retrieved from each treated explant, and each biopsy was analyzed using RT-qPCR. There were therefore 3 data arising per treatment for this latter study: 1 for each of the 3 biopsies taken from each of the 5 treated explants, for this single donor case. The data arising are summarized in Figure 3.

The chemexfoliation products were tested at the supplied concentrations recommended for clinical use. Histological examination of H&E-stained sections revealed that tissue integrity was maintained over the 14-day culture period (Figure 1c), where the general appearance of the skin upper layers is retained and largely unchanged from day 1 through day 14 on an untreated control sample. Inspection of Figure 1d illustrated that treatments using TCA-based chemexfoliants showed a moderate reduction in the number of epidermal cells with increasing TCA concentration, together with a corresponding slight reduction in epidermal thickness. Mild parakeratosis was also observed in treated samples, with expansion of the stratum corneum. The strongest treatment (20% TCA) showed reduced (or absent) stratum corneum thickness; however, this may be due to excess desquamation and loss of cornified cell layers in the fixation process. To further quantify bioeffects arising as a result of the chemexfoliant application and also facilitate direct comparison with the literature, we examined the regulation profiles of several distinct genes (forming type 1 collagen and also elastin), selected for their intrinsic importance in supporting the natural structural integrity of the skin and also as indicators of dermal remodeling in response to chemexfoliation treatment. In this latter context, the formative response of hyaluronic acid (HA) acts as a driver for keratinocyte proliferation and motility as a natural wound-healing response to external insult. Time points were chosen to exploit the maximum reliable culture period of the model (ie, 14 days). In addition, inflammatory response was evaluated 3 days after treatment, a choice that was informed by earlier preliminary time-course studies that showed peak expression of inflammatory markers at that particular time point (data not shown).

Collagen and elastin are 2 key dermal extracellular matrix (ECM) components that form networks of fibers responsible for the tensile strength and elasticity of the skin. These support matrices are often highlighted as indications of healthy and youthful skin (Reilly and Lozano, 2021), the content of which within skin declines gradually with aging. The data presented in Figure 2 highlight how 4 key biological markers respond to the various chemexfoliation treatments applied to each ex vivo skin model. The overlayed statistical analyses within each graph in Figure 2 compare post-treatment measurements on the retrieved biopsies (with RT-qPCR) with those of their respective untreated control samples. Elevated mRNA expression of *COL1A1*, a gene that encodes the pro-alpha chains of type I collagen secreted by dermal fibroblasts, was apparent in the TCA-based chemexfoliant–treated samples (Figure 2 [leftmost inset]) compared with that of untreated controls. Indeed, there is a highly significant (at least a 15-fold) increase in *COL1A1* present in the 20% TCA-treated skin after 14 days compared with that of untreated controls, with correspondingly lower but statistically nonsignificant responses observed with the milder chemexfoliants that employed lower TCA content levels.

Elastin is an additional key protein component of the ECM in the skin. It is highly elastic and is present in connective tissue, allowing many tissues in the body to resume their shape after stretching or contracting. Elastin in humans is encoded by the *ELN* gene. We noted that changes in expression of *ELN* (Figure 2 [second inset from left]) were more varied, with no definite trend observed as a function of each chemexfoliants’ TCA concentration; however, significantly increased expressions were observed in all treated samples, again relative to those of the 3 untreated controls from each donor.

Another chief ECM component implicated in the general condition of the skin and which is a function of the aging process is HA. HA influences keratinocyte proliferation and migration, and owing to its hydrophilic nature, it regulates water balance and stabilizes structures within the dermis. HA is synthesized by localized HA synthases (*HAS1*, *HAS2*, and *HAS3*), each producing distinct isoforms differing primarily by polymer size (Buhren et al, 2020). Regulation of these synthases can be influenced by many factors, including response to wounding (Papakonstantinou et al, 2012). More specifically, it has been reported that *HAS2* mRNA can be upregulated by IL-1b and TNFα in fibroblasts (Wilkinson et al, 2004). Notably, we observed in this study that all treated samples exhibited significant changes in *HAS2* expression (Figure 2 [second from right]). Interestingly, however, *HAS3* expression only exhibited a significant change in samples treated with the 20% TCA peel (Figure 2 [rightmost]), leading to strong downregulation.

**Figure 4:**
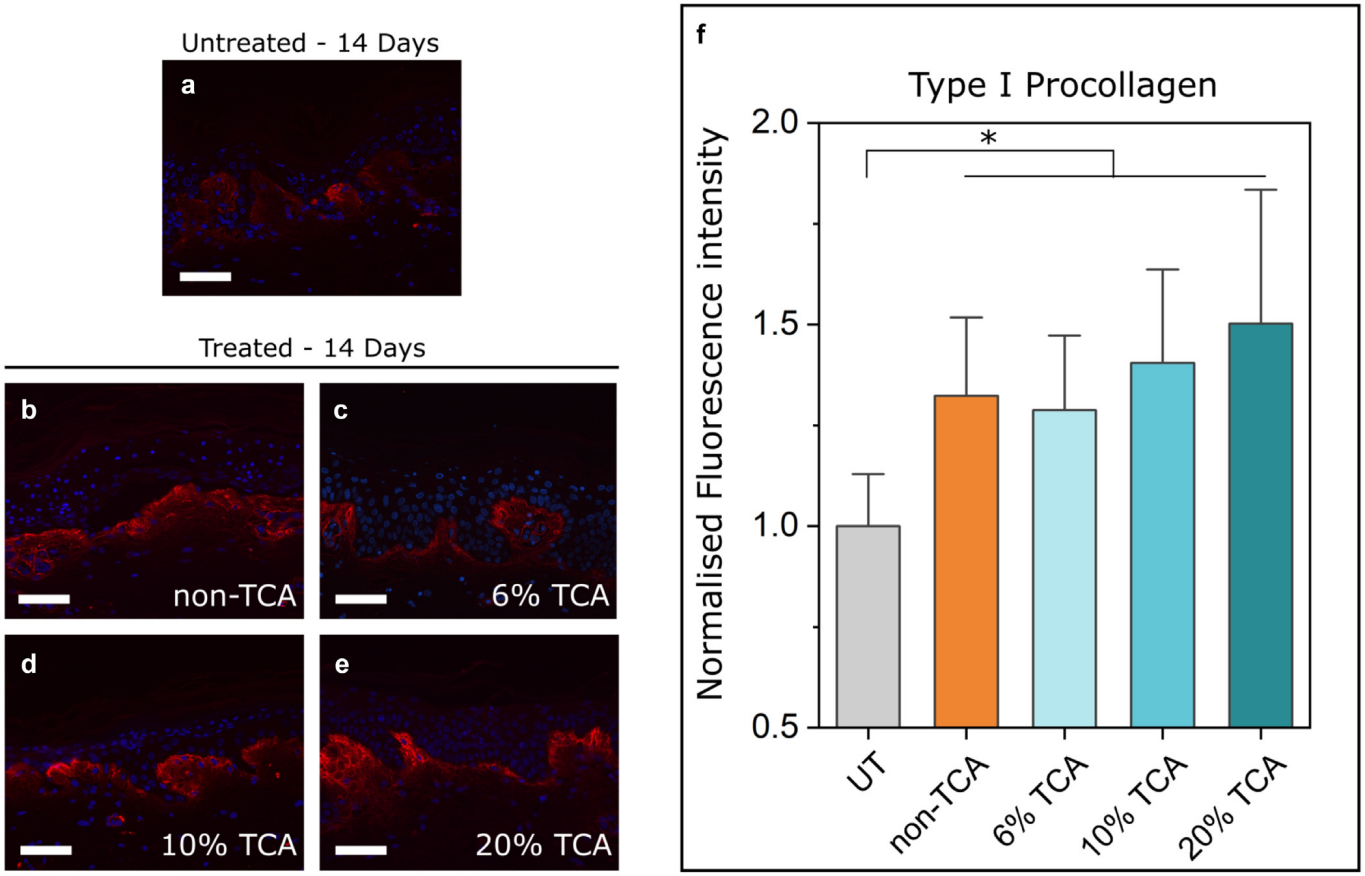
**Procollagen type I immunofluorescence staining.** Comparison of immunofluorescence staining intensity in (**a**) UT control and (**b**−**e**) peel-treated samples after 14 days in culture. Note that nuclei are stained blue (with DAPI), whereas procollagen I appears red. (**f**) Relative quantified fluorescence intensity calculated using mean pixel values in threshold mask areas delineating procollagen type I fluorescence signal. Significance is displayed in the graph (∗*P* ≤ .05) as determined by a 1-way ANOVA. The data are presented as mean ± SD from 3 image regions from a single donor (female aged 43 years). Bars in **a–e** are 50 μm. UT, untreated.

With benchmarked responses now established for 3 different TCA-based treatments upon 3 different individual donors (Figure 2), we proceeded to assess and evaluate the response of the recently developed non-TCA formulation. In this study, we used explant skin from a new individual female donor (aged 43 years) who had not been one of the 3 participant donors in the earlier benchmarking study (summarized in Figure 2) and whose data arising are presented in Figure 3, Figure 4.

Interestingly, application of the non-TCA agent resulted in a significant and positive expression profile response 14 days after treatment, matching or exceeding at least 1 of the TCA-based treatment responses for each ECM marker observed. Inspection of Figure 3 shows a >5-fold increase in *COL1A1* (Figure 3a [leftmost inset]) that is comparable with the 10% TCA treatment response for this particular experiment and with the responses to all TCA-containing agents in reasonable agreement with the benchmarked data represented earlier in Figure 2 (leftmost inset).

The *ELN* response (Figure 3a [second inset from left]) indicates an approximately 3-fold increase relative to that of the untreated control, which is comparable with the general response for the TCA-containing agents in this experiment. However, we note that this apparent change is not statistically significant under the 1-way repeated measures ANOVA analysis. Noticeably, in comparison with the benchmarked data for *ELN* shown in Figure 2 (second inset from left), the overall responses measured here are somewhat suppressed, again underscoring our general observation that *ELN* measurements appear to exhibit the greatest donor variation. The *HAS2* response to the non-TCA agent (Figure 3a [third inset from left]) is seen to be significantly upregulated relative to that of the untreated control and the 6 and 10% TCA-containing agents but less than the 20% TCA agent in this circumstance. Direct comparison with the *HAS2* benchmarked data (Figure 2 [second inset from right]) shows that these additional (Figure 3) preliminary data also compare favorably for the 6% and 10% TCA agent responses. The *HAS3* response for the non-TCA agent (Figure 3a [rightmost inset]) also shows a comparable downregulation to the 6 and 10% TCA agents, with the data here again also in reasonable agreement with the earlier benchmarked data illustrated in Figure 2 (rightmost inset).

In addition to the panel of ECM markers, distinct early inflammation responses were observed 3 days after treatment (Figure 3b). Proinflammatory cytokine IL-1b, which plays key roles in the initial inflammation phase of wound healing (Xiao et al, 2020), was significantly elevated in the strongest (20% TCA) treatment and to a lesser extent in those with the lower TCA concentrations. Most interestingly, the non-TCA agent–based treatment exhibited no significant elevation in IL-1b. We thus speculate that this formulation may be less prone to the inducement of adverse responses in clinical contexts. To qualify this inference, it should be further noted that upregulation of inflammatory cytokines has also been associated with the induction of matrix-degrading enzymes such as matrix metalloproteinase 9 (Ichihashi et al, 2009), suggesting that the >32-fold increase in IL-1b observed in the 20% TCA–treated skin samples could indicate the initiation of a more pronounced dermal remodeling process that does not occur with milder chemexfoliation treatments. Indeed, such an inflammatory response might also influence the strong downregulation of *HAS3*, as observed in our measurements, where low-molecular-weight HA fragments, such as those synthesized by *HAS3*, can have proinflammatory effects (Bracke et al, 2010).

Finally, we undertook measurements of extracellular procollagen (Figure 4), a standard approach to gauging the precursor collagen formation that is indicative of dermal remodeling. Consistent with the earlier gene expression results (Figure 2, Figure 3) and our interpretation presented earlier, immunofluorescence staining showed correlating elevation of type I procollagen protein within the superficial dermis across all treated sampled 14 days after treatment.

Chemical peels involve the application of a chemical exfoliant to the skin to disrupt the bonds that hold dead cells to the surface of the skin. Various types and strengths of chemical peels effectively wound the skin to various degrees and depths, effecting a dermal remodeling response that ultimately leads to (varied) improvement of the texture and appearance of the skin. TCA-based treatments remain commonly used in chemexfoliation procedures given their proven performance and safety profiles. When formulated with additional active components such as lactic acids, synergistic modes of action, including inhibition of ECM component enzymes and induction of genes responsible for ECM component synthesis, have recently been reported (Bhardwaj et al, 2021). These complementary actions can work to both protect native ECM and stimulate rejuvenation while maintaining skin barrier integrity and minimizing inflammation. Nevertheless, even with well-managed pretreatment and post-treatment care, there remains a risk of complications, although often transient, in patients with sensitive skin types or differing melanin contents. Advances using innovative chemexfoliation treatments (such as the non-TCA–based formulation evaluated in this study) may promise improved risk–benefit profiles and a lower barrier to entry, providing added comfort to first-time chemical peel patients.

As chemical peel formulation technologies continue to evolve, robust in vivo relevant preclinical testing methods should form an important part of the development process to ensure both efficacy and safety. The ex vivo model and methodology presented in this paper, although clearly not capturing the full complexity of in vivo tissue and its intrinsic biological response(s), may still inform usefully on aspects of the chemexfoliation-driven responses occurring in the skin. Maintenance of tissue integrity to 14 days after application was observed and furthermore returned meaningful data over a wide spectrum of commercial chemexfoliation agent strengths. An additional caveat to the data and inferences presented in this paper relates to the anatomical region from which skin was harvested to form all of the explants used. We used abdominal skin exclusively as opposed to the usual target area for chemexfoliation treatments, which is predominantly facial skin. This point notwithstanding, our observations suggest that the ex vivo model utilized in this study provides a robust and informative platform to investigate the efficacy of active ingredients and treatment regimens with the possibility to benchmark explant tissue response against chemexfoliation formulations with known in vivo performances. We advocate the protocol developed in this study as a highly useful means of benchmarking tissue responses to chemexfoliation agents that already have some clinical track record of use (and a reasonable prior understanding of biological action) against future experimental formulations so as to achieve indicative performance responses prior to transitioning toward clinical studies. Previously, degradation of tissue integrity over time in traditional in vitro and other ex vivo models has often necessitated the dilution of commercial chemexfoliation formulations to complete studies, which we believe reduces the overall relevancy. Evaluating potential new treatments in real human skin also offers an advantage to potentially extend downstream study coverage to embrace optimized chemexfoliation treatments across all 6 Fitzpatrick skin types and thus offers the possibility for later developing bespoke and individualized treatments that are intrinsically optimized to diverse skin types.

Our faith in the potential of our protocol is bolstered by the additional data represented in Figure 4, which illustrate that procollagen was significantly upregulated across all treated samples, which we infer to be consistent with new dermal remodeling occurring within each sample. Coupling this insight with the upregulation of *HAS2* and *Col1A1* measurements (Figure 3) underscores that the explant model responds to treatment in a manner consistent with the promotion of dermal remodeling.

To summarize, we have found that the TenSkin explant accomplishes the study objectives, with responses scaling against TCA concentration in commercially available products in a meaningful and intuitive fashion and with tissue integrity maintained out to 14 days, which matches clinically useful endpoints. We conclude that the establishment of this protocol represents a particularly useful means for initial testing of chemexfoliation formulations in a scientifically robust and reliable way that could usefully inform downstream preclinical trials.

## Materials and Methods

### TenSkin ex vivo model production

**Figure 5:**
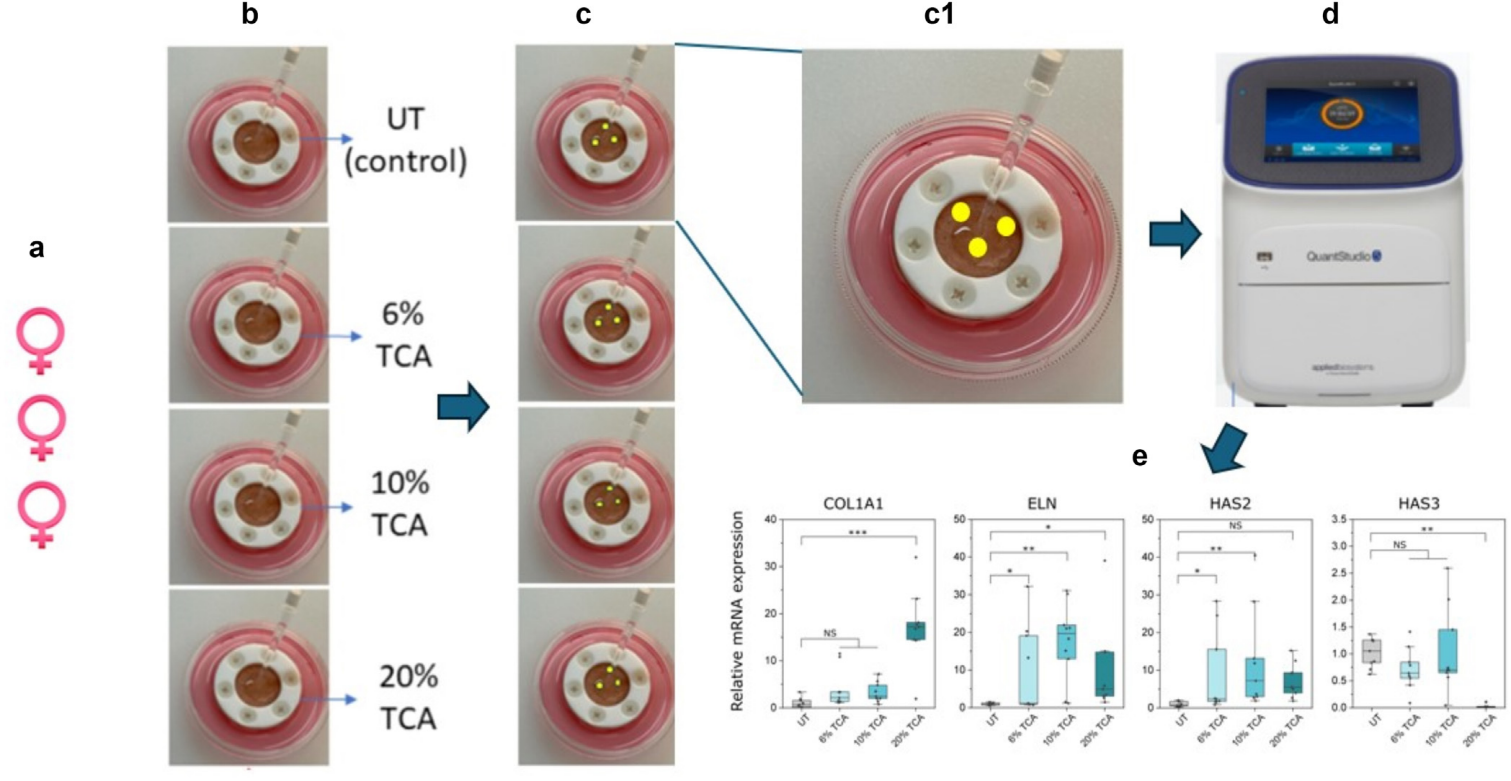
**Workflow summary.** (**a**) For the initial benchmarking study, the results of which are summarized in Figure 2, there were 3 White female donors aged 42, 43, and 59 years. (**b**) Each donor’s skin was then used to make 4× TenSkin units (viz Figure 1a), which were then treated with 6, 10, and 20% TCA chemexfoliants, with 1 UT control also implemented for each donor. (**c**) Fourteen days after treatment, 3 × 3 mm biopsies were taken at random locations upon each of the treated and control (UT) TenSkin units. (**c1**) An enlarged image of the TenSkin unit with 3 × 3 mm biopsy locations indicated (yellow circles). Note that 3 biopsies were taken from each unit to take account of any local structural inhomogeneities at the biopsy sites. (**d**) RT-qPCR was then used to measure each individual biopsy sample. (**e**) The statistically analyzed data presented in Figure 2 are reproduced in this figure to illustrate that from the RT-qPCR measurements arising, each of the 4 treatment regimens (UT and 6, 10, and 20% TCA) thus generated 9 data points within each box and whisker plot. TCA, trichloroacetic acid; UT, untreated.

Human tissue was obtained from elective abdominoplasty procedures with donor consent under Pearl institutional review board approval in accordance with Food and Drug Administration 45 CFR 46.102 and 21 CFR 56.102 regulations (Pearl Pathways, Indianapolis, IN) (exemption determination submission, institutional review board study number: 21-TENB-101, study title: Collection, culture, and distribution of human abdominoplasty skin tissue). Written informed consent was obtained from all subjects. All donors were healthy, and no identifying information beyond ethnicity, sex, and age was provided. The donors in the benchmarking study (summarized in Figure 2) were female of Fitzpatrick phototypes I and II and aged 42, 43, and 59 years, respectively. The additional donor for testing the non-TCA agent was also a White female aged 43 years. Tissue was transported and stored between 4 °C and 8 °C prior to processing for tissue culture (between 2 and 6 hours after surgery). Subcutaneous adipose tissue was removed from the skin samples, and the remaining full-thickness skin (epidermal and dermal layers) was washed for 10 minutes twice in PBS containing 2× antibiotic antimycotic solution (Sigma-Aldrich, A5955). Tissue regions in close proximity to surgical incisions or exhibiting defects such as edema or heavy striation were discarded. TenSkin models were assembled using a proprietary process to restore the physiological mechanical tension into the skin tissue. This mechanical tension was maintained by custom plastic culture frames creating a viable exposed epidermal surface area of 2.54 cm^2^ (18-mm diameter) viz Figure 1a. Any residual moisture remaining on the skin surface was removed by gently dabbing with sterile Kimwipes. The base of the frame was 3-dimensional printed with biocompatible acrylonitrile butadiene styrene, and the top of the frame was milled from polytetrafluoroethylene, which is also recognized for its biocompatible properties. Once assembled, TenSkin models were cultured at the air–liquid interface in standard 6 cm tissue culture dishes containing 7.5 ml of optimized serum-free cell culture medium (containing 2× antibiotic antimycotic solution), which was changed every 24 hours (Figure 1b). TenSkin models were maintained in humidified incubators at 37 °C and 5% carbon dioxide. The overall workflow is summarized in Figure 5.

### Chemexfoliant administration

Topical treatments were performed by pipetting 175 μl of the respective peel product to the center of the TenSkin sample (Figure 1b), which covered the entire exposed skin surface. No specific degreasing step was included prior to this application; however, negligible surface grease appeared present after the tissue washing steps were completed during the ex vivo model preparation. After the defined treatment time (15–30 minutes), the TenSkin sample was rinsed in fresh PBS 3 times to wash the peel product away and placed in fresh culture medium. Residual liquid or moisture was removed by gently dabbing with a Kimwipe before the TenSkin model was returned to the incubator.

### Harvest

To evaluate mRNA levels, three 3-mm biopsies were harvested from 1 half of each TenSkin explant, placed into collection tubes before being snap frozen in liquid nitrogen, and stored at −80 °C until further processing. To preserve the tissue morphology, the remaining skin, after biopsying, on each TenSkin platform was fixed at the optimized culture tension in 10% neutral buffered formalin at room temperature for 48 hours before processing for histological analysis.

### Histological analysis

After formalin fixation, skin samples were washed twice with fresh PBS and removed from the plastic culture frames and then dehydrated with increasing concentrations of ethanol to xylene, followed by embedding in paraffin wax. A total of 5 μm cross-sections were mounted on glass slides (Leica BOND Plus, S21.2113.A) and deparaffinized and hydrated for either standard H&E staining or fluorescent immunostaining of procollagen type 1. Briefly, for fluorescent immunostaining, Proteolytic-Induced Epitope Retrieval was performed with trypsin (Abcam, ab970) for 15 minutes at room temperature, followed by blocking with Abcam Protein Block (Abcam, ab64226) for 30 minutes at room temperature. Sections were then incubated in antiprocollagen type I primary antibody (Merck, MAB-1912) diluted at 1:50 in antibody diluent (Abcam, ab64211) for 2.5 hours at room temperature in a humidified chamber. After multiple washes, the sections were incubated in an Alexa Fluor secondary antibody (Abcam, ab150158) at 1:1000 in antibody diluent (Abcam, ab64211) overnight at 4 °C. Slides were finally washed and mounted with hydromount containing DAPI at 1:5000.

### RNA isolation and RT-qPCR

Total RNA was extracted from skin biopsies after mechanical disruption and homogenization (4 minutes at 30 Hz; 5 mm metal bead in RLT buffer) using the Tissue Lyser (Qiagen). Total RNA was isolated from the biopsies using a RNeasy Plus 96 Kit (Qiagen, number 74192), following the manufacturer’s instructions, and stored at −80 °C. cDNA was synthesized from ∼100 ng of total RNA using the High-Capacity cDNA Reverse Transcription Kit (Applied Biosystems, number 4368813), following the manufacturer’s instructions, and stored at −20 °C. RT-qPCR was performed using a Quant Studio 5 Real-Time PCR System (Applied Biosystems) and the corresponding TaqMan Real Time qPCR Assays (Applied Biosystems) to quantify relative expression (ΔΔCt) of selected genetic markers. *RPS13* mRNA was used as the endogenous control for all qPCR experiments.

### Data and statistical analysis

Data plotting and statistical analysis were done using OriginPro 2022b (OriginLab, Northampton, MA). One-way and 2-way repeated measures ANOVA was used to determine the statistical significance between group means (∗*P* ≤ .05, ∗∗*P* ≤ .01, and ∗∗∗*P* ≤ .001) determined relative to their respective untreated controls.

Relative quantified fluorescence intensity analysis was done using ImageJ (National Institutes of Health, Bethesda, MD). Relative procollagen type I fluorescence signal was quantified by applying a global threshold and subsequent mask to segment image regions with positive signal over background. Mean pixel values within the segmented regions were normalized to the area of each respective region and used as an arbitrary unit measurements of procollagen type I signal. Three images from each sample were analyzed and normalized to the mean of the untreated sample to provide a relative difference in fluorescence intensity across the samples.

## Ethics Statement

Human tissue was obtained from elective abdominoplasty procedures with donor consent under Pearl institutional review board approval in accordance with Food and Drug Administration 45 CFR 46.102 and 21 CFR 56.102 regulations (Pearl Pathways) (exemption determination submission, institutional review board study number: 21-TENB-101, study title: Collection, culture, and distribution of human abdominoplasty skin tissue). Written informed consent was obtained from all subjects.

## Data Availability Statement

The datasets analyzed in this study are available from the corresponding author upon reasonable request.

## ORCIDs

Michael J. Conneely: http://orcid.org/0000-0003-0212-1757

Jin Namkoong: http://orcid.org/0000-0001-8162-4453

Francis Allison: http://orcid.org/0009-0002-4053-3728

S. Kyoko Hirata Tsutsumi: http://orcid.org/0009-0002-6630-8561

Dominic Grussu: http://orcid.org/0009-0006-2411-4979

Ryan Willis: http://orcid.org/0009-0002-0780-2746

Kyle Henderson: http://orcid.org/0009-0006-3917-806X

Paul A. Campbell: http://orcid.org/0000-0001-9417-4586

Ewelina Lesniak: http://orcid.org/0009-0006-4196-7770

Mel Moy: http://orcid.org/0009-0001-6880-1811

Joanna Wu: http://orcid.org/0000-0003-2815-6851

Robyn P. Hickerson: http://orcid.org/0000-0002-7196-3694

## Conflict of Interest

Ten Bio is a for-profit company focused on the development of advanced ex vivo skin models, including the TenSkin model. JN, JW, EL, and MM are employees of Colgate Palmolive. Study chemexfoliation formulations were provided by Colgate Palmolive. The remaining authors state no conflict of interest.
